# A qualitative Interview Study Investigating Patient, Health Professional, and Developer Perspectives on Real-World Implementation of Patient-Centered AI Systems

**DOI:** 10.21203/rs.3.rs-7908218/v1

**Published:** 2025-11-07

**Authors:** Natalie Benda, Pooja Desai, Zayan Reza, Victoria Winogora, Uday Suresh, Yiye Zhang, Alison Hermann, Rochelle Joly, Jyotishman Pathak, Meghan Reading Turchioe

**Affiliations:** Columbia University Irving Medical Center; Columbia University Irving Medical Center; Columbia University Irving Medical Center; Columbia University Irving Medical Center; Vanderbilt University Medical Center; Weill Cornell Medicine; Weill Cornell Medicine; Weill Cornell Medicine; Arizona State University; Columbia University Irving Medical Center

**Keywords:** Artificial intelligence, predictive algorithms, bioethics, patient-centered care

## Abstract

Our objective was to triangulate patient, health professional, and developer perspectives for implementing patient-centered artificial intelligence (AI) systems. We conducted semi-structured interviews with patients (N = 18), health professionals (N = 8), and AI developers (N = 8). We created interview guides informed by frameworks in bioethics and health information informatics. We utilized a predictive algorithm for determining risk for postpartum depression as a use case to concretize our discussions. Our team analyzed transcripts from interview recordings using thematic, directed content analysis and the constant comparative process. Participants found mitigating potential harms caused by AI (e.g., bias, stigma, or patient anxiety) greatly important. They also believed that AI must provide clinical benefits by allowing health professionals and patients to easily take actions based on AI output. To take safe action, end users needed transparency to understand the AI’s accuracy and predictors driving risk. Patient participants wanted health professionals to interpret AI output, but health professionals did not always feel they had the time or training to do so. Participants also raised concerns regarding how data quality may affect AI accuracy, who may be responsible for inappropriate actions taken based on AI, and issues regarding data security, privacy, and accessibility. Our results support real-world implementation of more patient-centered AI tools by: providing health professionals with competencies for discussing AI-based risks; engaging patients and health professionals throughout the development process; inclusively communicating AI output to health professionals and patients; and implementing multi-layer systems of AI governance.

## INTRODUCTION

The transition to artificial intelligence (AI) in healthcare continues to be significant for patients and health professionals.^1–4^ Despite AI’s predictive and generative power, many systems are never implemented or, when implemented, struggle to show improvements in patient outcomes compared to the standard of care.^5^ This clinical translation gap between AI development and real-world, successful deployment is defined as the “last mile” problem. The “last mile” problem is the fact that AI models may show high accuracy and promise in simulated lab-based tasks, but (1) they are either never deployed in clinical environments, or (2) they cannot improve outcomes in real-life due to deficiencies in implementation. A critical gap lies in end users seeing, accepting, and acting upon AI-based recommendations.^6^ Previous research in human-AI interaction has focused heavily on health professionals as the sole recipients of AI recommendations, with less attention to implementing patient-centered AI systems.^7^ Our previous pre-implementation work for AI-based systems highlighted the importance of keeping patients informed regarding AI-informed recommendations to justify interventions and to support uptake.^8^

Patients and the public have important perspectives regarding AI implementation and want to be part of the conversation.^9–11^ However, their perspectives on centering patients in AI system implementation have been understudied compared to health professionals and AI developers.^7,8,12–17^ Moreover, previous studies have independently surveyed patient, health professional, and developer preferences about AI implementation. No studies to our knowledge, however, have collected richer qualitative data and combined their collective insights. Consequently, we lack integrative, practical guidance for those seeking to ethically implement and evaluate patient-centered AI systems.

It is also becoming more likely that patients may access AI output, even if not intended by AI developers. The U.S. 21st Century Cures Act, for example, prevents information blocking from patients, requiring organizations to give patients access to their electronic health information without delay or expense.^18^ This may result in a patient seeing a predictive AI risk score before discussing it with their healthcare team. AI is also increasingly used in tools to support shared decision-making with patients.^19^ While AI output may benefit patients, it may cause stress if not appropriately contextualized. AI can also be prone to hallucinations, the creation of false information, which may be challenging to differentiate from factual evidence.

Our objective was to triangulate perspectives from patients, developers, and health professionals for real-world implementation of patient-centered AI systems. We interviewed these three stakeholder groups using interview guides informed by bioethics and informatics models.^20^ To concretize discussions, we used an existing predictive AI tool developed by our team that proactively predicts a pregnant patient’s risk of postpartum depression (PPD) as a use case. Recruited patients and health professionals were potential users of the tool. PPD presents a complex bioethical case because of the sensitivity of the data and for the multi-layer autonomy; in pregnancy, the autonomy, harms, and benefits to the perinatal patient, newborn or fetus, and partner must be weighed simultaneously.^11,12^ Our study provides novel, data-driven recommendations for how AI may be ethically implemented to support patient-centred care.

## RESULTS

### Participant Characteristics

Table 1 provides demographic data from each stakeholder group (N = 36).

### Emerging themes

We inductively derived emerging themes for implementing patient-centered AI systems that cut across our overarching framework for analysis, Sittig and Singh’s Socio-technical Model for Studying Health Information Technology in Complex Adaptive Healthcare Systems (Table 2)^20^. We describe the detailed emerging themes with the most illustrative quotes from developers (DV), health professionals (HP), and patients (PT).

### Mitigating harm

All participant roles stressed the importance of ensuring minimal harm to end users (e.g., patients, health professionals) related to AI. One primary source of harm discussed involved stigmas driven by AI-based predictions. For example, predicting risk for mental health conditions or addictions (e.g., opioid use disorder) could change patient care trajectories. Patients expressed fear that risk predictions of mental health issues could lead to child protective services (CPS) interventions.

*I may run a 100 score and it’s like, ‘oh i passed’. Like, why is the doctor coming to talk to me? And ACS* (local CPS department) *is coming to take my child?*

- PT12

Participants also raised the concern about model bias, noting the importance of utilizing representative training data and valuing fairness equally to accuracy.

I’m sure they’d be curious if their ethnicity is put in there…But what if there is some type of bias in the tool?

- HP02

Patients and health professionals viewed AI as potentially harmful due to the stress that high-risk predictions may cause patients. Participants described the importance of involving health professionals and patients in the early stages of AI development to identify and mitigate these harms.

The patient is going to develop that insight and be on board… and lead to like a more … patient-centered treatment.

- DV08

### Clinical benefit and utility

All groups expressed that AI must provide clinical benefits to patients, care partners, health professionals, or health systems. Participants agreed that for models to provide utility, a health professional or patient must be able to easily take action (e.g., making a referral, prescribing a medication, contacting their provider) based on AI’s output. Patient and health professional participants seemed most comfortable with AI that could “run in the background” and provide support to human decision-makers as needed.

If it can explain its reasoning, I feel like that’s more helpful…I like to think of AI like as a tool. So if it was actually like teaching me and…I can learn what those risk factors are and take the time, then I would find it way more useful.

- PT16

For the tool discussed (predictive AI for PPD), patient participants were typically open to their providers using the tool as an additional data point and accepting treatment based on the AI and their doctor’s input. However, one patient was very averse to using the tool, indicating the preference-sensitive nature of AI use in clinical care.

I would be confused because I wouldn’t believe that they could figure that out…it seems so bizarre. I probably would lose a bit of trust.

-PT09

Translating predictive models into action also requires a threshold for action. Patient participants, however, had challenges understanding what the numeric risk predictions meant practically and why there were certain thresholds for action (e.g., the tool recommended a mental health referral if the PPD risk was > 30%).

0 to 30%, you know, that’s a closer gap. And then you have like this gap from 32 to 100, but it’s like 40, 50, 60, 70. And I’m assuming the prevention treatment is starting at either 75 or 80. And I was like, what about those people in the middle?

- PT12

To trust AI and promote relevant actions, most end users wanted to understand the accuracy of AI. Ideally, patients wanted to know how accurate it was for them individually. However, some patients did not need to see this information themselves but trusted health professionals to make judgments on when AI was safe for clinical use.

I assume that [if] it’s in my patient portal, I’m thinking that it’s accurate for me.

- PT01

Developers noted a tension between high performance and making understandable models that people wanted to use.

Interviewer: *Accuracy versus understandability and explainability, and if you could only choose one, which one would you choose and why?*

DV01: *We need both, you can’t really do it without both, but when forced…the majority of the crowd choose understandability*.

Participants described different strategies for ensuring models provided benefits. First, health professionals need to support clinical validation and interrogate potential biases. Participants also highlighted the need to investigate and plan for how AI may alter existing workflows. Updates would then need to be implemented in line with institutional norms and policies.

Developers viewed implementation and ensuring utility as a continuous process that would require updates over time, similar to any traditional software. Developers also acknowledged the importance of end-user feedback throughout AI model development. Although they did not always feel they had the skills to include this feedback, particularly related to patient users.

I think one piece that’s definitely missing in my research is patient perspectives about whether they think this kind of tool would be useful…I think that those questions have to be asked very carefully…But I don’t know that developers should be the ones actually going in to ask those questions.

- DV07

### Communicating AI Models

Several questions emerged regarding if, when, where, and how AI-related information should be communicated to patients. Patients had differing perspectives regarding how they would like to view AI output, with some preferring to see it before clinical encounters and others wanting someone to explain it to them. Multiple participants agreed that patients would need support in interpreting information and suggested that the provider may be the best person to do this. However, they also noted that the provider may need training and dedicated time to do this effectively.

Who gets the alert, and what happens once you get an alert? If you’re going through all that, then how much time are you spending to explain it?

- HP06

Others highlighted that interfaces should be presented in lay terms, accessible to different audiences. Feature importance was discussed at length - participants suggested conveying predictors of risk (e.g., medications, history of past illness) as both positives and negatives contributing to the patient’s total risk. Patients generally wanted to see all features, not only the modifiable predictors. Providing sensitive features (e.g., risks related to marital status, BMI) requires care but may stimulate educational conversations. It was also important to ensure the labels were understandable by patients, so using the EHR-based feature names (e.g., “thyroid preparations”, “hyperemesis”) may be insufficient.

Patients also reported varying opinions on their desire to see AI-based risk information. Some felt they had a right to see these risks, how accurate they were, and what their individual risk factors were. Others trusted their provider to see the information and make the related decisions.

### Data quality

Participants described challenges related to ensuring data quality, primarily related to bias, missingness, out-of-date information, and shortcomings in interpreting unstructured data (e.g., clinical notes) with insufficient context.

These days…charts are not well updated… unless it’s thoughtfully updated, which it rarely is because hospital systems are far too busy for that kind of meticulousness, then I think that people are going to feel like I could see people being like, ‘where the hell did that come from?’

- HP03

Missingness was noted to be particularly problematic in a perinatal context, where many may receive care for other specialties in different health systems.

### Data security, privacy, and access

Participants agreed that the way the data is stored, the algorithm is computed, and displayed to end users must protect patient and health professional privacy. There was, however, a tension between making tools open source and protecting patient privacy, ensuring their autonomy over their data.

Many viewed external organizations, such as the Centers for Medicare and Medicaid, as having a responsibility for ensuring privacy (i.e., data de-identification) and also governing privacy rules, although it was not always clear as to which entity was responsible. Health organizations were also mentioned as having a role in supporting data privacy and promoting data access. There were other external organizations whose access to data (e.g., CPS/ACS) was viewed as detrimental.

Patients had different desires for accessing AI inputs and outputs, including through the patient portal, on mobile phones, via PDF, and secure email. Some also wanted the ability to share the information with chosen care partners, but this varied from person to person.

Others highlighted how the combination of perinatal and psychiatric concerns made privacy issues more sensitive. Some patients, for example, may be reluctant to disclose sensitive information due to stigma or fear, leading to data accuracy issues.

Whenever a pregnant woman talks about this type of information…that concerns like calling to Child Protective Services. That is a big privacy issue…clinicians worry about models raising severe alerts.

- DV09

Participants working in pediatrics also noted that accessing maternal health information through a child’s chart can be challenging due to limitations in data linkage capabilities and due to confidentiality protections in the EHR for sensitive maternal data.^21^

### Responsible AI practices and liability

Participants had various opinions about who was responsible for the safe development and implementation of AI. Participants mentioned responsible entities at various levels from insurers and device manufacturers to individual developers and health professionals.

There needs to be some responsible party who’s well informed, who’s making the decisions about if and how these tools are actually used and up to date on how well they’re actually being used in patient care as you introduce them

- DV08

Many of the questions of responsibility stemmed from a desire to mitigate individual liability for health professionals. The prevailing perspective was that no single person or entity could be held responsible, and a structure of shared responsibility was necessary. Some perceived that determining safety for the use of an algorithm should fall to health professionals. Health professionals also highlighted the need for responsibility in terms of seeing and acting upon risk predictions. Developers, on the other hand, felt responsible for making systems with high predictive performance that also mitigated potential biases.

So a clinician needs to make an informed decision. In which case…the clinic is liable, as they would be for any misdiagnosis or incorrect treatment decision.

- DV08

If we implement it then…somebody has to have ownership to it that you are responsible for this. And if the patient is seeing this information, somebody has to interpret for them.

- HP06

Responsible practices mentioned included checking for bias and accuracy prior to implementation, as well as continuous monitoring to identify model drift and retrain models. The conversation surrounding ensuring accuracy could be challenging, however, as the gold standard for comparison may not always be clear.

## DISCUSSION

AI implementation in health systems requires coordination between patients, clinicians, developers, and health systems administrators and leaders. Through interviews with nearly 40stakeholders representing these perspectives, we identified requirements implementation and use of patient-centered AI systems. We summarize our findings in preliminary requirements derived directly from stakeholders, shown in Table 3. Our results specific to end user perspectives on conveying AI-based risk of PPD overlap with Williams et. al’s recent qualitative study on a similar topic.^22^ However, our work extends upon their findings, incorporating developer perspectives and using PPD as a use case to make broader recommendations for patient-centered AI regardless of clinical context. Our findings also resonate with the recently released AI Code of Conduct for Health and Medicine from the National Academy of Medicine, related to: advancing benefit for humanity, ensuring equity, engaging impacted individuals, ensuring workforce wellbeing, monitoring performance, and supporting continuous learning.^23^ We describe practical next steps with data-driven insights for achieving these requirements across key stakeholder groups.

First, health professionals will require competencies to use AI effectively and safely in healthcare.^24^ Most patients reported that they wanted their health professionals to deliver predictive AI results, whereas most clinicians do not feel qualified to do so. Health professionals also highlighted the difficulty in interpreting AI output and the limited time during encounters. While some efforts have attempted to integrate AI competencies into medical and nursing education,^24^ far more work is needed to educate the existing workforce of health professionals.

Second, ur findings highlight how decisions made during the development process can have downstream impacts, for example, balancing explainability with accuracy, or addressing fairness. Model developers, at times, work in relative isolation from the health system leadership, clinicians, and patients who may ultimately adopt their model. Technical development is viewed as a foundational step with little input needed from other stakeholders. Rather, our findings suggest that because of the downstream implications of decisions made at the modeling stage, health professional and patient stakeholders should be included in the early stages of development and evaluation to ensure models prioritize the right values, are useful, and ethically sound.

Participants also raised issues related to how data completeness and timeliness may affect model accuracy. Similar to displaying significant model features, visualization may support end users in understanding when key data elements may be missing or outdated. A systematic review of 44 implemented predictive AI models highlighted the lack of attention to user workflow and providing predictive risks in a way that enhances rather than degrades cognitive processing.^12^ Future work may investigate generalizable best practices for displaying predictive AI models to work collaboratively with human end users.

Third, to provide clinical utility, predictive AI output needs to be actionable, as we have previously reported.^8^ For example, perinatal providers need a way to refer appropriate patients for psychiatric or social work evaluation. Participants noted several needs to promote actionability, including providing reasoning (i.e., risk factors) and the level of model accuracy. This information would allow them to weigh their expertise in conjunction with the model, and choose from multiple possible actions based on the patient’s risk factors. Patients wanted to understand individual risk factors, which could support shared decision-making conversations with providers regarding risk reduction. Participants also noted that meaningful thresholds for action based on the numeric risk prediction were needed. This is not done consistently in practice, and there is a lack of robust methods for determining these thresholds for actionability.

Fourth, patients in our study expressed a wide range of preferences and information needs related to interpretation of the model. Compared to clinician end users, less attention has been given to patients as end users of models. There are many scenarios in which patients may view model output, making their perspectives vital in considering how to display output. The harms of failing to incorporate their perspectives may include elevated worry and anxiety, increased outreach to care teams, and even unnecessary care visits.

Lastly, several other publications have provided detailed needs assessments for enhancing privacy, navigating liabilities, and devising regulations related to AI.^23,25–28^ Our findings reinforce that layers of shared governance, involving multiple stakeholder groups, will be necessary to address these challenging questions.

Our findings also suggest multiple important areas of future work. First, our study primarily focused on predictive AI models. Generative AI is being rapidly adopted in research and in healthcare settings. A recent systematic review identified over 200 studies that have used large language models (LLMs) for healthcare purposes, including patient education, recommendations, and guidance, customizing care plans, interpreting medical information, and facilitating patient-clinician communication.^29^ They reported positive findings relating to patient satisfaction but concerns about readability, accuracy, and bias. Thus, investigating patients’ needs and ability to use LLMs safely will be an important part of ensuring appropriate use in healthcare.

Additionally, the science for both predictive and generative AI has weighed heavily towards model development and validation, with significantly fewer studies evaluating real-world clinical implementation and health outcomes. Multiple perspective pieces have called for more implementation science studies,^30^ along with clinical trials that have thoughtful comparator arms and benchmarks representing the current state of the healthcare system.^31^ While improving clinical outcomes is the ultimate goal for most predictive AI systems, recent studies have highlighted that only measuring these outcomes may produce an incomplete picture, particularly when AI does not have the intended effect.^32^ We advocate for detailed tracking (e.g., via EHR log data) that may ascertain how AI impacts decisions, how it is accessed by patients, and the downstream impacts this may have on patient care. All of these metrics may not be assessed in a detailed manner, but having them available supports investigating “last mile” problems when highly accurate AI fails to improve clinical outcomes. This evidence is critically needed to inform continued surveillance and refinement of AI models and to guide strategies for operationalizing the broad guidelines and frameworks that professional bodies such as the National Academy of Medicine have published.^23^

Finally, there are notable gaps in patients’ expressed interest in understanding more details about model predictions, and the strategies that we presently have to provide this information in a clear and comprehensible way. Despite substantial interest in the explainability of AI, very little explainability work has considered patients as end users.^33^ Clinicians themselves may also struggle to determine how and when to follow AI recommendations, let alone how to deliver this information to patients.^34,356^ These findings point to the need to both equip health professionals, including nurses and providers, with AI-related competencies or “literacy.”^34,3524^

Limitations of this study include that we based part of our interview guide on an existing algorithm predicting postpartum depression; thus, findings may not generalize to other clinical contexts or populations. Additionally, though we strived to invite a diverse range of stakeholders to participate in interviews, some perspectives may have been underrepresented. Thus, future work building upon these findings to further investigate patient-centered AI across a range of clinical contexts and populations, with additional stakeholder groups, is needed.

## CONCLUSION

Patients, health professionals, and AI developers provided important insights on AI implementation that considers patients as active end users. Participants described how to mitigate potential harms related to AI, how AI may provide meaningful benefits, recommendations, and new questions for communicating AI models, as well as challenges related to data quality, privacy, security, access, and liability. Based on our findings, we provide recommendations for patient-centered AI implementation that include engaging stakeholders throughout development (not just at the end), supporting health professionals with competencies for interpreting and communicating AI risks, and inclusively communicating AI in ways that also support taking relevant clinical actions.

## METHODS

### Ethics approval

All participants provided written informed consent. The Columbia University Institutional Review Board approved the study.

### Setting, Study Design, and Sample

We conducted prospective qualitative interviews with AI developers, health professionals, and perinatal patients. Health professionals and patients were potential end users of the tool in our use case, and AI developers had relevant expertise but were not directly involved in development of the given tool. Table 4 describes the inclusion criteria and recruitment methods.

### Data Collection

**Figure 1:**
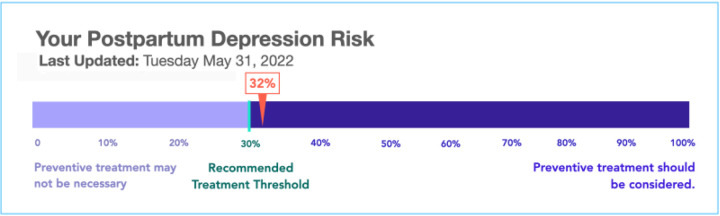
Sample output of postpartum risk prediction AI tool shown to patient and heath professional participants.

Our team iteratively developed interview guides (Supplemental file), combining insights from MITRE’s Ethical Framework for the Use of Consumer-Generated Data in Health Care and Sittig and Singh’s Socio-technical model.^20,36^ Experts in obstetrics, perinatal mental health, nursing, AI development, consumer informatics, and qualitative methods provided input. We piloted each guide before enrollment. We showed patient and health professional participants a mockup of how the algorithm from our use case (i.e., proactive prediction of postpartum depression) may be displayed to concretize the discussion (Fig. 1). The mockup was informed by a survey of over 500 perinatal participants.^37^ We found that participants trusted AI-based risk scores that included a concrete risk number (instead of phrases like “above average”), and that participants preferred number line graphics over numbers alone.

Three researchers trained in qualitative methods conducted all interviews via Zoom or telephone (NCB, PMD, ZR). Interviews lasted 30–60 minutes and were audio recorded. Participants were compensated $50. Following the interview, participants electronically completed a demographics survey.

### Data Analysis

We conducted a directed content analysis using thematic analysis and the constant comparative process.^38^ In this paper, we focus on insights derived from the dimensions of the Sittig and Singh Socio-technical Model, which we first used to deductively organize the data, but with adapted definitions to suit our application to predictive AI.^20^ A group of three coders (NB, PD, ZR) first analyzed transcripts collaboratively. Two team members then coded each transcript independently. We then met to compare and resolve disagreements, using the third coder in cases where agreement could not be reached. We also reviewed transcripts for thematic saturation, ensuring our sample met this criterion based on the diminishing need to update code definitions. Once all transcripts had been coded, we reviewed the data code by code to mitigate code drift. One of the original coders (NB) and another team member (MRT) then reviewed each parent code to derive emerging themes that cut across the domains of the Sittig and Singh framework.

## Figures and Tables

**Table 1:** Participants’ demographics provided at the aggregate level and broken out by stakeholder group.

Demographic characteristic		Stakeholder Group			
		Overall (N = 36), n(%)	Developers (n = 10), n (%)	Health Professionals (n = 8), n (%)	Patients (n = 18), n (%)
**Sex**	Female	29 (81)	5 (50)	7 (87.5)	17 (94.5)
	Male	4 (11)	4 (40)	0 (0)	0 (0)
	Missing	3 (8)	1 (10)	1 (12.5)	1 (5.5)
**Race**	White	13 (36)	5 (50)	2 (25)	6 (33.5)
	Asian	6 (17)	4 (40)	2 (25)	0 (0)
	Black or African American	9 (25)	0 (0)	2 (25)	7 (39)
	Native Hawaiian or Other Pacific Islander	1 (3)	0 (0)	0 (0)	1 (5.5)
	More than One Race	2 (5.5)	0 (0)	0 (0)	2 (11)
	Prefer Not to Answer	2 (5.5)	0 (0)	1 (12.5)	1 (5.5)
	Missing	3 (8)	1 (10)	1 (12.5)	1 (5.5)
**Age (generation)**	Generation Z (1997–2012)	6 (17)	3 (30)	0 (0)	3 (17)
	Millennials (1981–1996)	23 (64)	5 (50)	5 (62.5)	13 (72)
	Generation X (1965–1980)	3 (8)	0 (0)	2 (25)	1 (5.5)
	Boomers (1946–1964)	1 (3)	1 (10)	0 (0)	0 (0)
	Missing	3 (8)	1 (10)	1 (12.5)	1 (5.5)
**Education**	Some College or Associate’s Degree	6 (17)	0 (0)	0 (0)	6 (33.5)
	Bachelor’s Degree	11 (31)	1 (10)	2 (25)	8 (44)
	Master’s Degree	8 (22)	2 (20)	3 (37.5)	3 (17)
	Doctorate Degree	8 (22)	6 (60)	2 (25)	0 (0)
	Missing	3 (8)	1 (10)	1 (12.5)	1 (5.5)

**Table 2:** Emerging themes from our analysis and their intersection with the dimensions of Sittig and Singh’s Sociotechnical framework.

	Sittig and Singh Dimension							
Emerging theme	Software, hardware, and computing infrastructure	Clinical content	Human-computer interface	Workflow and communication	People	Internal organisational policies, procedures and culture	External rules, regulations and pressures	System measurement and monitoring
**Mitigating harm**	X	X		X	X		X	X
**Clinical benefit and utility**	X		X		X	X		X
**Communicating AI models**	X	X	X	X	X			
**Data quality**	X	X		X				
**Data security, privacy, and access**	X				X	X	X	
**Responsible AI practices and liability**					X	X	X	X

**Table 3:** Stakeholder-driven requirements for patient-centered AI.

Topic	Requirement description	Related themes
Health professional competencies related to AI	Provide health professionals with the time and training to deliver AI-based risk information	Mitigating harm, communicating AI, responsible AI practices
Ethical creation of AI systems	Engage health professionals and patient stakeholders throughout the development process	Mitigating harm, clinical benefit and utility
Effective communication of AI output for all stakeholders	Promote actionability by displaying options for viewing model accuracy, predictors, and thresholds for action	Mitigating harm, communicating AI, responsible AI practices
Patient-centered AI communication	Include flexible options for communicating AI output to patients to manage cultural preferences and varying literacy levels	Mitigating harm, communicating AI, responsible AI practices
AI governance	Making ethical decisions related to governance issues, including privacy, access, liability, and regulation, will require multiple levels of stakeholder engagement	Data security, privacy, and access; responsible AI practices and liability; AI regulation

**Table 4:** Inclusion criteria and recruitment sources by participant stakeholder group.

Stakeholder group	Inclusion criteria	Recruitment sources
AI developers	Involved in the AI technical development process Had experience with AI applications in mental or reproductive health English-speaking	Professional organizations (e.g., the American Medical Informatics Association), and personal connections
Health professionals	Provides care to perinatal persons (related to somatic care, mental health care, or both) in the U.S. English-speaking	Departmental listservs, personal connections
Patients	Currently pregnant or within one year postpartum Received/receiving perinatal care in the U.S. English-speaking	Social media (Instagram, Reddit, Facebook), Columbia University (RecruitMe)

## Data Availability

All raw data is available for sharing upon reasonable request to the corresponding author.
